# “You Can Lead a Horse to Water, but You Can’t Make It Drink”. Gastroenterology Healthcare Professionals’ Perceptions of Reasons for Patient Non-Adherence to Inflammatory Bowel Disease Prescribed Medication: A Qualitative Study

**DOI:** 10.2147/PPA.S569308

**Published:** 2026-04-03

**Authors:** Kathryn King, Christine Norton, Awa Jammeh, Trudie Chalder, Wladyslawa Czuber-Dochan

**Affiliations:** 1 Florence Nightingale Faculty Nursing Midwifery and Palliative Care, King’s College London, London, UK; 2 Department of Psychological Medicine, Institute of Psychiatry, Psychology and Neuroscience, King’s College London, London, UK

**Keywords:** inflammatory bowel disease, medication adherence, medication concordance, qualitative study, interviews, healthcare professionals’ perspectives

## Abstract

**Purpose:**

Inflammatory bowel disease (IBD) is commonly treated with medications to induce and maintain remission. Yet many patients do not take treatments as prescribed. Understanding healthcare professionals’ (HCPs) beliefs about medication non-adherence may help clarify whether their views align with patients’ experiences and inform future adherence support strategies.

**Patients and Methods:**

Semi-structured interviews were conducted with 21 purposively selected HCPs from diverse professional backgrounds working within gastroenterology in the National Health Service (publicly funded healthcare system in the United Kingdom). Interviews were video recorded, transcribed verbatim, and analyzed using Braun and Clarke’s principles of reflective thematic data analysis.

**Results:**

Four main themes were identified: 1) HCPs’ perceptions of patients’ adherence, including perceived reasons for adherence and non-adherence, honesty about adherence, sub-group differences, and the language used in consultations. 2) how HCPs seek to promote adherence through empathy, reassurance, provision of information, practical tools, support strategies, and treatment decisions aligned with patients’ needs. 3) challenges for supporting adherence, including everyday practice barriers, systemic constraints, and reflections on whether HCPs themselves may contribute to the problem. 4) what is needed to improve adherence, encompassing support for patients, HCPs, and healthcare systems, as well as proposed intervention approaches.

**Conclusion:**

HCPs recognize that adherence in IBD is shaped by multiple determinants, many of which can be influenced through clinical practice and patient interactions. Strategies such as empathy, reassurance, clear communication, reliable information, medication-taking tips, are believed to support adherence whilst providing safe practice. However, challenges arise from patient-, professional-, and system-level factors. Addressing these requires shared responsibility between clinician and patient, adequate consultation time, improved service capacity and sustained person-centered care. Flexible and timely training for HCPs may also enhance adherence support. Capturing HCPs’ perspectives offers valuable insight into medication adherence in IBD and can inform strategies to strengthen adherence support within clinical care.

## Plain Language Summary

Many people with inflammatory bowel disease (IBD) do not take their medication as prescribed. This is called non-adherence. Understanding the reasons for this may help to improve the support patients receive, which could lead to better disease control, less wasted medication, and reduced healthcare costs.

Twenty-one healthcare professionals (HCPs) from different professional backgrounds currently working in gastroenterology services were interviewed.

The findings highlight four main themes; 1) HCPs’ views on patients’ adherence and related behaviors; 2) how HCPs perceive themselves supporting people with IBD in taking medication; 3) barriers that make providing adherence support difficult; and 4) ideas for improving support in the future.

HCPs discussed many reasons why people take or avoid their medication. Some can be addressed through compassionate care that focusses on patient’s needs and wellbeing. Educating patients about IBD and treatments in clear, simple language was seen as essential for understanding and adherence. Patients also need to feel respected and involved in decisions about their care.

HCPs themselves can be better supported through teamwork, shared responsibility of adherence with both the team and patients, and access to training that is practical and feasible within busy services. Healthcare systems should allow enough time and capacity for HCPs to provide meaningful adherence support. In addition, technological tools, used alongside effective healthcare, may help people with IBD take their medication as recommended.

## Video Abstract

**Figure:**
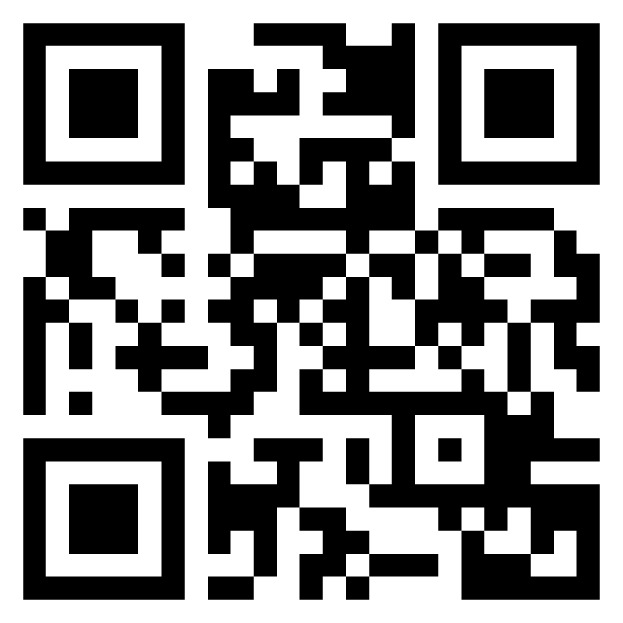

Point your SmartPhone at the code above. If you have a QR code reader the video abstract will appear. Or use: https://youtu.be/ThitVPoh1Ik.

## Introduction

Crohn’s disease (CD) and ulcerative colitis (UC), collectively known as inflammatory bowel disease (IBD), are chronic, incurable inflammatory conditions of the gastrointestinal tract. Symptoms vary from mild to severe and may include abdominal pain, bloody diarrhea, bowel urgency, weight loss, fatigue, and psychological comorbidities such as anxiety and depression.^1–3^

Pharmacological management of IBD typically follows a stepwise approach, progressing from aminosalicylates to corticosteroids, immunomodulators, and biologics until effective inflammation control and remission maintenance are achieved. Treatment success is highly dependent on adherence. Non-adherence in IBD is common, affecting up to 72% of patients, and is associated with more frequent and severe relapses, poorer disease control, and reduced quality of life (QoL). However, non-adherence is not unique to IBD, being a challenge across many chronic conditions.^4^ At a societal level, it contributes to increased healthcare utilization and costs.^5^

Despite this, evidence suggests that healthcare professionals (HCPs) often underestimate the prevalence and impact of non-adherence. More than half of gastroenterologists, trainees, and IBD nurse specialists report believing it to be infrequent.^6^

Physicians are poor at identifying patients with low adherence, correctly recognizing only one-third of such cases.^5^ Accuracy is much higher for identifying adherent patients.^5^ This under-recognition limits opportunities for intervention.^7^,^8^ Although most HCPs acknowledge that improving adherence would lead to better outcomes,^6^,^9^ adherence is not routinely assessed.^10^ When medication is reviewed, review is often prompted by treatment failure or recurrent relapse and typically undertaken by less experienced clinicians. Moreover, methods used, such as unstructured patient interviews are unvalidated and are frequently unreliable subjective adherence assessments.^5^

Evidence demonstrates that 99% of HCPs acknowledge that improving adherence would positively impact health outcomes in IBD.^6^ Identifying low or non-adherence and the reasons for it, are considered key to improving adherence, and improving patient health.^5^,^6^,^9^

Barriers to adherence are complex and multifactorial, encompassing patient, treatment, HCP, and health system factors.^4^,^11^ They may be intentional or unintentional, and modifiable or not.^11^ Common patient-reported barriers include poor disease or treatment understanding, medication side-effects or side-effect concerns, difficulties with regimen complexity, limited medication access, and forgetfulness. Psychological factors such as depression, anxiety, and negative illness and treatment beliefs also play an important role.^11^ Few studies have explored HCPs’ perspectives on adherence, despite evidence of gaps between patient and clinician views on symptoms, treatment priorities, and disease impact.^12^ Understanding HCPs’ beliefs about medication non-adherence in IBD is therefore critical to improving support strategies, aligning perspectives, and ultimately enhancing adherence and outcomes.

This study aimed to explore HCPs’ perceptions of the reasons for intentional and unintentional non-adherence to prescribed medications in people living with IBD. Objectives were to: i) obtain detailed insights into HCPs’ experiences of prescribing and monitoring IBD medications; ii) identify perceived factors influencing patient adherence and HCP engagement in adherence support; iii) elicit recommendations for strategies to promote adherence, targeting both patients and HCPs, and to inform the development of future adherence interventions.

## Materials and Methods

### Study Design

This qualitative study employed in-depth, one-to-one semi-structured interviews.

### Participants

We aimed to recruit approximately 20 HCPs working in the United Kingdom's (UK) National Health Service (NHS) gastroenterology or IBD services. Recruitment was conducted online though Crohn’s and Colitis UK (CCUK), Bowel Research UK, the Royal College of Nursing IBD Network (via Facebook), and snowball sampling. Eligibility criteria included being a qualified HCP (gastroenterologist, IBD nurse, pharmacist, dietitian, psychologist, psychiatrist), employed in an NHS gastrointestinal service for at least six months. Purposive sampling ensured diversity in professional background, length of service, hospital and clinical role, and demographics,^13^ with efforts made to capture a wide range of perspectives on medication adherence in IBD.^4^

### Data Collection

Interviews were conducted via Microsoft Teams between June and November 2023 using a piloted topic guide (Supplementary Figure 1), informed by prior systematic and scoping reviews.^4^,^11^ The guide covered healthcare roles, prescribing and monitoring responsibilities, perceptions of adherence and non-adherence, observed adherence behaviors, and support strategies and barriers. All interviews were audio recorded, transcribed verbatim by a professional transcriber, and anonymized prior to analysis. All interviews were conducted by KK, who had no prior relationship with participants. Field notes and a reflexive journal were kept. Recruitment continued until apparent data saturation was reached.^14^

### Data Analysis

Data familiarization involved repeated reading of transcripts and review of recordings.^15^ Reflective thematic analysis (RTA) followed Braun and Clarke’s framework,^16^,^17^ using primarily inductive coding,^18^ supplemented by deductive coding informed by researcher experience and existing literature. Coding was undertaken in Excel and with paper-based methods. Initial open coding generated emerging themes (eg, “non-adherence normalization”, “adherence honesty”), which were refined iteratively into broader themes and sub-themes.^18^,^19^

**Figure 1:**
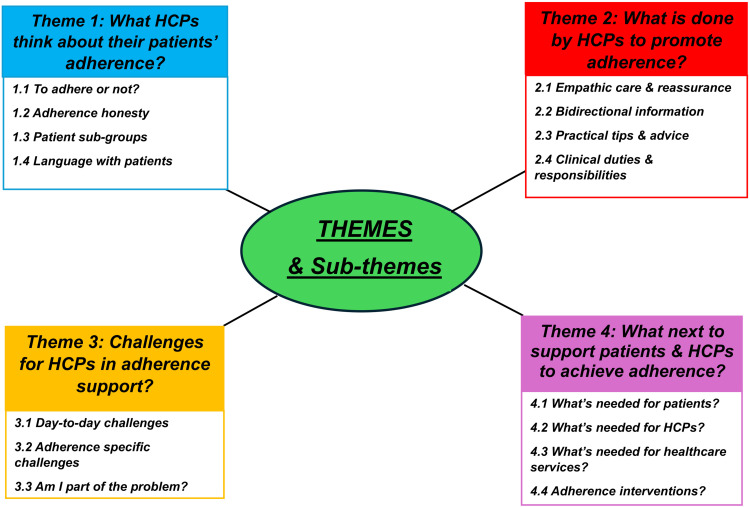
Themes and Sub-themes. Figure 1 outlines the four main themes and accompanying sub-themes identified.

**Abbreviation**: HCPs, Healthcare Professionals.

**Figure 2:**
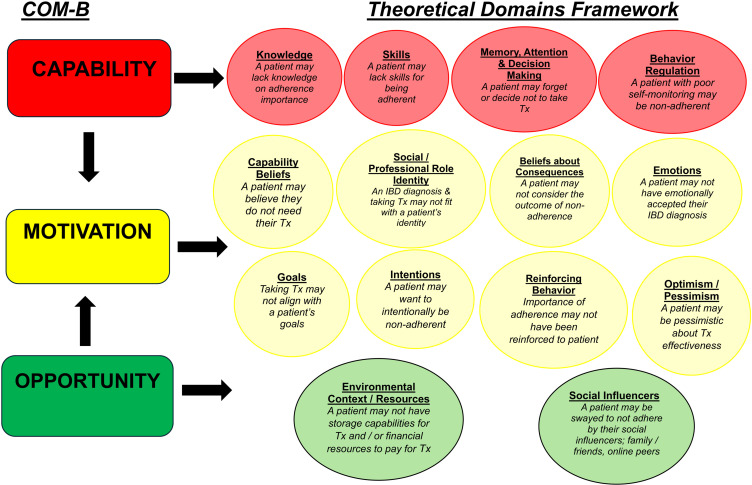
The Theoretical Domains Framework and COM-B model. Figure 2 relates the COM-B model and the Theoretical Domains Framework to example reasons for medication non-adherence in IBD patients, from the perspective of a Healthcare Professional.

**Abbreviation**: IBD, Inflammatory Bowel Disease; Tx, Treatment.

**Figure 3:**
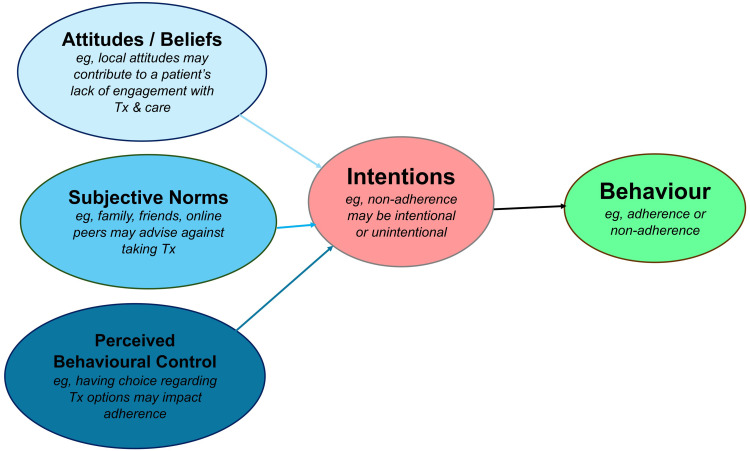
The Theory of Planned Behavior model. Figure 3 relates the Theory of Planned Behavior model to explain reasons for medication non-adherence in IBD patients, from their perspective.

Second level analysis aggregated codes into major themes (Figure 1). Following theme creation, these were mapped to established models of behaviour,^16^,^17^,^20^ including the:

- Theoretical Domains Framework (TDF)^21^ to review HCPs’ perspectives,
- COM-B,^22^ to analyze HCPs’ behaviors and
- The Theory of Planned Behavior (TPB)^23^ to explain patient behaviors (Figures 2 and 3).

### Trustworthiness

Rigor was addressed through confirmability, credibility, dependability and transferability.^15^ Reflexivity was embedded throughout data collection and analysis to mitigate potential bias.

The RTA group comprised researchers with expertise in qualitative methods, nursing, psychology and neuroscience, alongside one individual living with IBD, to strengthen authenticity and ensure representation. All coders received training and support from the lead researcher (KK).

Each transcript was independently coded by at least two researchers. KK and AJ coded all transcripts, supported by three supervisors (WCD, TC, CN). Discrepancies were resolved collaboratively in team meetings. In total, 624 initial codes were identified and refined into themes and sub-themes.

### Ethics

Ethical approval was granted by King’s College London Research Ethics Committee (HR/DP-22/23-34676). Written informed consent was obtained from all participants prior to interview, which included publication of anonymized responses/direct quotes. Anonymity and confidentiality were maintained through allocation of participant numbers. Participants could withdraw at any time and request withdrawal of their data within two weeks post-interview, although no one chose to do so. The study was conducted in accordance with the Declaration of Helsinki.^24^

## Results

### Recruitment

Forty-one individuals expressed interest in the study and received a participant information sheet. Three were ineligible. Thirteen did not complete the eligibility questionnaire or consent form. Of the remaining 25 eligible participants, one was not invited due to purposive sampling saturation. Twenty-four were invited. One did not attend and two cancelled, leaving twenty-one participants completing interviews. All interviewed participants consented to optional video recording. Interviews lasted between 48–84.5 minutes (mean duration: 64 minutes). Total interview time was 22.5 hours.

### Socio-Demographic and Clinical Data

Table 1 summarizes participant characteristics. The majority were consultant gastroenterologists or IBD nurse specialists, with additional representation from pharmacists. Two-thirds of participants were female. Most held either a master’s degree or research doctorate. Clinical experience ranged from 6–36 years (mean 18.6 years), with IBD-specific experience ranging from 2–30 years (mean 13.2 years). The majority were employed in teaching hospitals, with a minority based within general hospitals.

**Table 1:** Participants’ Professional and Demographic Characteristics

Items	Mean (Range) or Number
**Professional background**	
Consultant Gastroenterologist	6
Gastroenterology Specialist Registrar	1
IBD Nurse Specialist	5
Gastroenterology Nurse Consultant	1
Advanced Clinical Nurse Practitioner	1
IBD Specialist/Lead Pharmacist Gastroenterology	3
Consultant Psychiatrist	1
Consultant Psychologist	1
Gastroenterology Dietician	2
**Sex**	
Female	14
Male	7
**Highest Academic Qualification**	
Undergraduate	3
Postgraduate	18
**Years in practice since qualifying**	18.6 years (6–36)
**Years working with IBD patients**	13.2 years (2–30)
**Place of work**	
Teaching hospital	18
General hospital	3
**New IBD patients seen per week**	
1–3	11
4–6	7
7–9	0
≥10	3
**Follow-up IBD patients seen per week**	
1–5	2
6–10	5
11–15	6
16–20	3
21–25	1
≥26	4
**Percentage of weekly time spent in clinical practice**	
30–50%	7
51–70%	3
71–90%	7
91–100%	4
**Percentage of weekly time in IBD clinical research**	
<10%	12
10–30%	5
31–50%	3
51–60%	1
**Percentage of weekly time in other clinical research**	
0%	15
1–10%	6

**Abbreviation**: IBD, inflammatory bowel disease.

Regarding clinical workload, participants typically managed 1–3 new IBD patients per week and 6–15 follow-up patients. Over half reported spending ≥71% of their working week in clinical practice, while less than 10% of their time was dedicated to IBD research or other clinical research.

### Themes

Four main themes and associated sub-themes were identified (Figure 1). These are described below, supported by verbatim quotes (Table 2), referenced by participant details eg, P (participant number), M/F (male/female), and profession.

**Table 2:** Themes, Sub-Themes and Example Quotations Reflecting Healthcare Professionals’ (HCPs’) Perspectives on Adherence to IBD Medication

Themes & Respective Quotation	Sub-Themes and Respective Quotation	Example Quotations
1) **What HCPs think about their patients’ adherence?** “**It’s not as bad as it sounds [be]cause most people will be taking it most of the time. It’s just that odd one, missing the odd dose”**. (P18, M, IBD Consultant Gastroenterologist).	1.1) **To adhere or not?** “**I think in general most patients will come to the decision to take them”**. (P6, F, IBD Nurse Specialist).	**Diagnosis** a) “I do have a few patients that admit that they really find it difficult to take tablets, just to remember. I had a patient the other week telling me that it’s there by her toothbrush and yet it’s like she’s become blind to it and she still forgets to take it”. (P6, F, IBD Nurse Specialist). b) “Or they may be saying that they haven’t taken it, or they’ve got side effects because they don’t want to take it”. (P9, F, Gastroenterology Nurse Consultant). c) “They [patients] don’t want to take their injection when they feel well and all of that... I feel very well so I’m not going to take my medication”. (P2, F, IBD Nurse Specialist). d) “Normally I don’t think it’s [swallowing] something that is too much of an issue”. (P6, F, IBD Nurse Specialist). e) “I think it’s the effectiveness of the drug. I think if a patient has seen a really great improvement then they keep taking it. If they do not see any improvement or if they just feel like they are not getting anywhere then they do not want to go for the infusion, they do not want to take the drug”. (P5, F, IBD Nurse Specialist). f) “You’ll always get some patients that really just struggle to adhere to medications as much as they will want to try their lives are maybe too chaotic”. (P6, F, IBD Nurse Specialist).
	**1.2) Adherence honesty** **“People tell me they take their medications, but I know that in reality a lot of people don’t take their medication. So, I don’t know if people are always being 100% honest with me about taking it”**. (P6, F, IBD Nurse Specialist).	a) “I suppose it’s what you are like, if they are intimidated by you or they are worried about what you’ll think then maybe they would not be so honest”. (P1, M, Consultant Gastroenterologist). b) “I was like “Are you taking all your medications? It’s important that we know you are taking them. If you’re not taking them, tell me, because that might explain it”. He’s like, “Yeah, I’m taking them all, Doctor”. The nurse comes up and then literally 20 minutes later, he tells her, “Oh yeah, I wasn’t taking that yellow one. That really important one”. I saw him and said “Why did you tell me you were taking them when you were not taking them? And he was like “Ohh, I was afraid””. (P14, F, Consultant Gastroenterologist). c) “Be honest with me. …If you’re honest with me, there’s no consequences”. (P15, M, Consultant Gastroenterologist). d) “We ask… before we increase doses, we just need you to be honest with us. because what we don’t want to do is increase the dose and then overload you”. (P25, F, Lead Gastroenterology Pharmacist).
	**1.3) Patient sub-groups** **“You can lead a horse to water, but you can’t make them drink and we’ve done everything for them and they’ve just not turned up or they’ve not engaged”**. (P16, F, Consultant Gastroenterologist).	a) “I reckon young people are less likely to be adherent than older people. And they’re perhaps more likely to have slightly more moving parts and chaos and stuff than someone who is generally older…” (P1, M, Consultant Gastroenterologist). b) “I think the adolescent transitioning patient is probably the most difficult one in terms of adherence”. (P10, M, Consultant Gastroenterologist). c) “…They’re so good with their injections. I have to say the elderly are just amazing when it comes to compliance”. (P5, F, IBD Nurse Specialist). d) “When you talk about IBD in the elderly depending on what other comorbidities you have and the pill burden, adherence to certain pills will drop off depending on which you see as the priority amongst your six medical conditions you’ve got…” (P10, M, Consultant Gastroenterologist). e) “We have a lot of farmers, and they are the most stoic bunch of people that you could ever meet. They will not come to hospital, they will not ask for help unless there is absolutely something seriously wrong”. (P7, F, IBD Nurse Specialist). f) “So those young males that are under the age of 25 who are single, a fairly non-compliant bunch of people in general”. (P10, IBD Nurse Consultant). g) “It’s really tough because at the end of the day they are either going to do it or they’re not [adhere to treatment]. Some patients I think you could give them all the support in the world and they’re still not going to do it”. (P5, F, IBD Nurse Specialist).
	**1.4) Language with patients** **“…I don’t think I’d use any of those words to patients themselves.They are my words. They’re our words, not their words”**. (P9, F, Nurse Consultant).	a) “…They wouldn’t know what I said if I was talking about adherence and concordance with them”. (P20, M, IBD Pharmacist). b) “But I mean, in conversations, I do not say adherence to patients, I would say are you taking your medications? I am not going to say, are you adherent with your medication? [laughs] That’s, it’s not really a patient-facing type of word, I think. I would ask them what they are taking and how, you know frequency and that kind of things…” (P24, F, IBD Dietician). c) “I think I prefer concordance to have a concord with us… we’re not bringing the patient on the journey with us. It’s their journey not ours. We’re the passengers…” (P4, F, Advanced Clinical Nurse Practitioner). d) “No, I hate it. No, I do not. I do not talk about it and I do not even talk about adherence…I think all those words have a kind of association with a negative consequence. So you are not concordant. You know you are a bad person…And I think those words imply wrongdoing and I do not think patients are wrong…” (P16, F, Consultant Gastroenterologist). e) “So there’s something paternalistic about all the language of compliance, concordance, and adherence, in which we’re saying, here’s what you should be doing and what are you doing and where’s there a difference here, that is your fault”. (P15, M, Consultant Gastroenterologist).
2) **What is done by HCPs to promote adherence?** **“Methotrexate Monday, Folic acid Friday”**. (P16, F, Consultant Gastroenterologist).	**2.1) Empathic care and reassurance** **“They’re much more compliant if we work with them rather than against them”**. (P5, F, IBD Nurse Specialist).	a) “Patients don’t fit into boxes”. (P4, F, Advanced Clinical Nurse Practitioner). b) “Be open with them to be able to make the best plan and be clear with them”. (P25, F, Lead Pharmacist Gastroenterology). c) “As well as understanding A) the disease course and B) the treatment you also have to understand the person because I’m never going to get A and B sorted unless I get them to take the treatments which involves understanding C) really”. (P10, F, IBD Nurse Consultant). d) “But you do therefore need to be available to sort of patients to contact you regarding what any problems they are having with side effects and that kind of thing to advise them and to change the treatment, which quite often has to happen. Yeah, so being available in the early stages to offer advice”. (P22, M, Consultant Psychiatrist).
	**2.2) Bidirectional information** **“No old-style leaflets no. If I want someone to look at a leaflet about Adalimumab or Azathioprine or Vedolizumab or Ustekinumab I’ll say look at the CCUK web, just Google Ustekinumab CCUK”**. (P1, M, Consultant Gastroenterologist).	a) “One of the things I am keen on is that is about trying to quantify those things like we were talking about cancer risk…if you can say, oh, there’s a rare risk of cancer. It’s much better, I will say, ‘You know the lymphoma risk is 2 in 10,000 in the general population and it goes up to 4 in 10,000,’ which is much more reassuring than it than saying, oh, there’s an increased risk of cancer. So, I am quite keen to do that when I can sort of use numbers with patients”. (P21, M, Lead Gastroenterology Pharmacist). b) “We talk about those paling palettes with the lots of men and then a couple of them are colored in and they’re the ones who have lymphoma on Azathioprine and ways to communicate risk…” (P1, M, Consultant Gastroenterologist). c) “We can come up with a plan around what next and then, but also the patient and I can then have the conversation around, OK, why aren’t you taking it? What? How do we troubleshoot this?” (P23, F, Consultant Psychologist). d) “I think you got to be careful that you say to a patient or don’t say to them early on this is it for the rest of your life because it may well not be”. (P16, F, Consultant Gastroenterologist). e) “The other thing as well is if you try to sell them a long-term treatment is maybe not to set off as a long-term treatment in the beginning. Let us give this a go and let us see what difference it makes to your symptoms over the next two months. Then they come back, and they go ‘oh my God it was life changing’”. (P10, F, IBD Nurse Specialist). f) “I’d probably just say to somebody are you taking this regularly or just in questioning... I say to somebody so are you taking your Asacol regularly? Yes, yes I am taking it. So, what dose? I do not try and trick people, but I would say so you are taking da, da, da. Yes, I am taking that. So, if I say so you are still taking your medication. And they will say yes. Just remind me which one do you take? OK so what dose do you take of that?” (P7, F, IBD Nurse Specialist). g) “Are you actually taking it?... Levels are showing you are not taking it”. (P25, F, Lead Pharmacist Gastroenterology).
	**2.3) Practical tips and advice** **“Alarms or the [medication] apps… Physically putting it somewhere, stick it in your knicker drawer or where your keys are - so that you literally have to trip over it”**. (P24, F, Gastroenterology Dietician).	a) “I think we always try and make it as simple as possible for them... injection at hospital every 8 weeks vs. injection at home every 2 weeks”. (P25, F, Lead Pharmacist Gastroenterology). b) ”As soon as you show them the pen [for self-administration] they go oh that’s fine. So yes once they see it and I think once they get round to OK yes this is OK... So I think once they see it and once they realise they do not have this giant needle they are quite happy with it, they are alright with it”. (P5, F, IBD Nurse Specialist). c) ”I have done that [encourage family and friends to help]…when people are not managing to self-inject and having to get friends and family to [help]. Yes definitely, yes it’s very powerful isn’t it(?), particularly if you are living with them”. (P13, F Gastroenterology Specialist Registrar). d) “Some people don’t really like technology. Dosette boxes and blister packs suggested when memory is a concern… or getting family, friends, carers involved can help as well”. (P25, F, Lead Pharmacist Gastroenterology).
	**2.4) Clinical duties and responsibilities** **“There’s a duty of care you need to let them know what you think the likely outcome and possible worst outcome that might be”**. (P13, F, Gastroenterology Specialist Registrar).	a) “If the patients are feeling well, they sometimes can’t see the point of [monitoring]... so we really talk about that in depth”. (P25, F, Lead Pharmacist Gastroenterology). b) ”We have not been great at doing the shared-care stuff where we and you know, [be]cause it it’s been a problem here. And then it was not done well historically. So you know, it’s something that we probably need to look at getting better at…nobody wanted to take on extra responsibility”. (P20, M, IBD Specialist Pharmacist).
3) **Challenges for HCPs in adherence support?** **“Medicine would be really easy if you have one patient and we would all be absolutely brilliant, not just doctors, but healthcare workers, if you had more time and less patients to look after”**. (P18, M, Consultant Gastroenterologist).	**3.1) Day-to-day challenges** **“We probably don’t have the time or resources to tackle it [adherence] as well as we could do really. I probably admit that I don’t routinely ask about adherence as well as I should do”**. (P21, M, Lead Pharmacist Gastroenterology).	a) ”We have a clinical psychology team and their waiting lists as you can imagine, are absolutely shocking because there’s such a need for them”. (P24, F, Dietician). b) ”So it’s not something that you can talk in the beginning of your journey and say right this is your first consultation, I am your consultant, I must emphasize that you must take your medications regularly and then you forget about it and then six months down the line no one has picked up on adherence, no one has even asked the question are you taking your pills? One year after that you are going to PIFU, you are disappeared into the ether, no one bothers”. (P11, M, Consultant Gastroenterologist). c) ”The age old war cry of you do not have time in clinic, you do not have time not to because actually every time we give the wrong drug or every time we give a drug with no information which the patient then does not take that’s time that we have wasted because they will have to come back to say that it did not work. So in actual fact we do not have time for that. We have to do it right the first time I think”. (P4, F, Advanced Clinical Nurse Practitioner). d) “So we use the helpline but it’s completely inundated so the nurses get loads, and there’s a pharmacist helpline as well so they get hundreds of emails every day about people asking about their medication”. (P1, M, Consultant Gastroenterologist). f) “When you ask patients if they have a personalized care plan they usually say no. And when you ask staff if they are providing personalized care they usually say yes. That’s because that understanding what that perception of personalization is giving patients time to ask their questions or plan questions in advance”. (P9, F, IBD Nurse Consultant).
	**3.2) Adherence specific challenges** **“But sometimes you do think I wish we could go back 30 years where they would just be like, well, the doctor said I should do it. So I’m just gonna do it because you’re like I know your life would be so much better”**. (P14, F, Consultant Gastroenterologist).	a) “And so you know, if it’s someone has a problem with injections and you are just swapping them from one injection-based med to another, it’s not gonna change anything. So yeah, just kind of introducing a bit of like can we have a discussion around this and type conversation to happen as well”. (P23, F, Consultant Psychologist). b) “I don’t know that we are very good at accepting it, acknowledging or listening to issues where actually it’s about adherence or drug choice. It’s very easy and doctors are much more they just escalate and escalate and actually you come back and actually the patient has never really taken the basics very well”. (P9, F, Nurse Consultant). c) “Struggling with trauma around a hospital admission or surgery and the need for a stoma, for example, that they are then blaming themselves because they didn’t appropriately take their medications for in the past and that yeah, that’s really tricky”. (P23, F, Consultant Psychologist). d) “You know it, it’s very, very difficult to convince a patient who is non symptomatic who is currently very well that they need to take their medicine? Yeah, I think that’s profession wide. I think that’s every speciality who has that problem, too, GPs, everyone. I know there’s nothing wrong with you, but I need you to take this”. (P20, M, IBD Specialist Pharmacist). e) “There’s always horror stories out there and I think there’s always somebody who knows somebody who knows somebody who had the worst thing happen to them imaginable which is why I am very careful about directing people to peer support”. (P2, F, IBD Nurse Specialist). f) “But the difficulty is that quite often the experiences that they are hearing are the negative ones because those are also the people who are looking for answers and looking for help... they will often hear those stories of the person that had an anaphylactic reaction or had the worst case scenario or that the drug did not work and it was the fourth one that they have tried. As opposed to the people who get on to a treatment that’s very effective and then they go off travelling or they go to university and then they do not want to talk about it because they do not feel the need to”. (P7, F, IBD Nurse Specialist).
	**3.3) Am I part of the problem?** **“I am hoping I am not normalizing it so much that they think it’s OK to not do it. So you sort of reinforce it, you know if you are just missing it, you know now and again that’s OK. But obviously if you miss it regularly, it is more problematic. I hope you are not going to talk to patients now and they say, oh, yeah, the doctor normalized it for me not to take medication. That’s not my intention. I had not really looked at it that way before. I do not think I normalize it that much”**. (P14, F, Consultant Gastroenterologist).	a) “I can tell you without question that it’s the behaviors and beliefs of the healthcare professionals are as important as the behaviors and beliefs of the patients they are looking after”. (P2, F, IBD Nurse Specialist). b) “You are not adherent. You are…and it and it. And actually it’s I do not like the way of speaking that makes somebody feel like they are a bad person or they have done something wrong. Actually we need, it’s a system thing and we need to help them become part of the solution”. (P16, F, Consultant Gastroenterologist). c) “Well if they said I miss it once in a blue moon or even once a month I would kind of say do not stress too much. If you are doing OK, your levels are OK and you miss it once in a blue moon what do you want to do? No one is perfect, everyone will miss it once in a blue moon. It’s just life”. (P1, M, Consultant Gastroenterologist). d) “There’s a few where, you have to chase and chase and chase, and part of that is because they’re not, you know, that they’re not coming in to have certain interventions so, I don’t have to ask because I know they haven’t even had it”. (P19, F, Gastroenterology Dietician). e) “So yes we do but if somebody is well on Mesalazine we just leave them to it. We leave them to it. And we assume that if you don’t get in touch with us that that medication is working for you”. (P5, F, IBD Nurse Specialist). f) “I think it’s a much bigger, I think it’s one of these things that we do not know what we do not know… we do not know the scale of the problem, and I think there’s loads to be kind of looked at in terms of how to engage with this [adherence] and work with this better”. (P23, F, Consultant Psychologist).
**4) What next to achieve adherence?** **“A drug is only as good as if the patient takes it or turns up for the treatment”**. (P10, F, IBD Nurse Specialist).	**4.1) What’s needed for patients?** **“I think mostly when it’s an unintended choice with enough reassurance and support, you can get over that hump”**. (P14, F, Gastroenterology Consultant).	a) “We have to, I think, change, how we do things slightly to be more available in those early stages because adherence is a big challenge”. (P22, M, Consultant Psychiatrist). b) “Generally it’s education and once people understand how the medication works then I feel like they take it”. (P6, F, IBD Nurse Specialist). c) “We have a flare card that we use that’s quite fundamental for those patients where they can have control over their medications where they can learn how to self-manage a bit better…it’s a card that you can share with everybody so all patients in our service with Crohn’s or colitis should get it. It talks about how you can escalate treatment if you feel you are getting symptoms…it allows them, it gives them permission to increase their 5-ASA dosing when they are flaring”. (P9, F, IBD Nurse Consultant). d) “So you know what, let us meet someone who’s had a difficult like, had some surgery or kind of this is another option. Let us have a chat or someone who’s on the medicine already. Let us chat about actually, it’s alright to come in for infusions, they are not that bad”. (P15, M, Gastroenterology Consultant).
	**4.2) What’s needed for HCPs?** “**I think it’s probably we could just do better with asking about adherence on a routine basis”**. (P21, M, Lead Pharmacist Gastroenterology).	a) “So I think a change of attitudes and beliefs which is the most difficult thing. It’s probably why it’s the thing [adherence] that sticks around”. (P2, F, IBD Nurse Specialist). b) “Everyone will have different styles and everyone is individual and people will respond differently to different people and that’s why we work as a team really”. (P5, F, IBD Nurse Specialist). c) “The pharmacist, the nurses that obviously the clinicians, consultants as well. Charitable bodies as well, like Crohn’s and Colitis UK. Yeah, I think they’d be very good at [adherence support] because they reach a lot of people in the support groups and things like that. And then. Obviously for specific patients, it’s, it’s carers and family members and things like that”. (P20, M, IBD Specialist Pharmacist). d) “I definitely think it’s an area where training is needed or would be needed. But I’m also not convinced that it would be engaged with all that well”. (P23, F, Consultant Psychologist). e) “But it’s a very practical job and when you are talking to patients you kind of want to know well how do I take that into practice though? It’s all well and good knowing that actually 60% of patients are not taking it but what do I do tomorrow in clinic if I see that somebody is not taking their medication? You want to know what are the points to raise with somebody, how do you raise it and what are the ways that you can educate patients I suppose”. (P7, F, IBD Nurse Specialist). f) “Having at least more than one session and having some sort of interval practical in your own clinic… and then people find out that actually there are all these problems that they would not have uncovered if they have not done that. That could be very, very powerful in that you are then suddenly seeing an impact on your practice and on your clinic because statistically, in one clinic of ten people you probably would find someone wouldn’t you if you asked everyone? Then having something where you fed that back and a shared experience about that, that could be really powerful rather than a one-off where you might not do that homework”. (P13, F, Gastroenterology Specialist Registrar).
	**4.3) What’s needed for healthcare services?** **“I’m quite protective about wanting to see our patients in a specialist service so that we can give them the best service regardless of how extensive or complex their disease is”**. (P4, F, Advanced Clinical Nurse Practitioner).	a) “Adherence is one small part of a big picture isn’t it(?) and I think really what they need is understanding and capacity to deliver personalized care planning of which adherence would be a part”. (P9, F, IBD Nurse Consultant). b) “There’s too many things to talk to somebody about in a clinic to do a kind of fully holistic assessment every time of every aspect”. (P9, F, Consultant Gastroenterologist). c) “Bounce ideas to improve medication adherence – have like peer review for more training upon this. I think the most important thing is that we are all working together... with the patient at the centre”. (P25, F, Lead Pharmacist). d) “Certainly for things like the biologics where they are just doing them every three months that’s really important about keeping a record of when your last one and all of those companies make handheld records for patients that they can use if they want to”. (P9, F, IBD Nurse Consultant). e) “It’s really hard. Because it’s all done through homecare we don’t really see them taking the medication we kind of just trust that they are”. (P5, F, IBD Nurse Specialist).
	**4.4) Adherence interventions? “A mixed media type thing of a bit of online app or app stuff and then having some check-ins with, a well-trained clinician and for support and follow up and would be a good way of doing it”**. (P24, F, Dietician).	a) “Something that’s app/web based where you can at least check alarms, rather than set an alarm on your iPhone you set an alarm on the app and it tells you and you have to press something and it says OK I have taken my medication or you can snooze it or say I am not going to take my medication today and then you get a visual representation of doses that are missed, taken, deferred, there can be reasons for not taking it, overdue prescription or whatever”. (P1, M, Consultant Gastroenterologist). b) “And the bottom line is people, really like talking to people”. (P18, M, Consultant Gastroenterologist). c) “I definitely see that there’s room for patient support like peer support, within patient groups. But it’s, it’s you know, getting like the right people and that people understand like you are not here to tell them all of their treatment, you are just here to kind of support them more, in a like general manner. I think it can be really good, but it can also go really wrong sometimes”. (P19, F, Dietician). d) “Something to keep people organized because life is busy and three months flies by without you even noticing it. So I think something like that would certainly be helpful for me if I had something like that. I think that’s the only thing that I can think of that would be good for patients to be able to do it. That would be amazing. Have I just found my million pound idea?” (P5, F, IBD Nurse Specialist). e) “Essentially it’s a three-step process of going from red, amber and green where you like, you are not ready for transition and then you are getting ready for transition and then you are ready for transition”. (P18, M, Consultant Gastroenterologist).

**Abbreviations**: CD, Crohn’s Disease; G.P, General Practitioner/Family Doctor; IBD, Inflammatory Bowel Disease; IBDU, Unclassified Inflammatory Bowel Disease; F, Female; M, Male; P, Participant; PIFU, Patient Initiated Follow-up; UC, Ulcerative colitis.

#### Theme 1: What HCPs Think About Their Patients’ Adherence?

It’s not as bad as it sounds [be]cause most people will be taking it most of the time. It’s just that odd one, missing the odd dose. (P18, M, Consultant Gastroenterologist)

This theme explored HCPs' perceptions of why people with IBD do or do not adhere to prescribed medication, and the extent to which patients are open about their behavior.

##### To Adhere or Not?

I think in general most patients will come to the decision to take them. (P6, F, IBD Nurse Specialist)

HCPs described adherence as a complex and dynamic, and individualized process, shaped by patient, treatment, and contextual factors (Table 2).

Most patients were perceived to accept treatment and be adherent, when the rationale was clearly explained, aligning with personal values, such as disease control and avoiding surgery (Table 2, Title-theme 1 quote). Securing patient “buy-in” at treatment initiation was considered essential for long-term adherence, involving clear explanations of necessity, risks, and benefits. Confidence and trust in the healthcare team, and motivation to improve health each promoted adherence (Table 2, quote 1.1). By contrast, non-adherence could be intentional or unintentional (eg, forgetfulness) and influenced by beliefs, mental health, poor understanding of disease or medication, and external influences (family, peers, online forums, lifestyle) (Table 2, quote 1.1a). Some patients preferred complementary and alternative medicine, highlighting the importance of accurate information to counter misconceptions.

Side-effects, both actual and anticipated, were considered highly influential (Table 2, quote 1.1b). Adherence was more likely when side-effects were minimal but decreased when patients were asymptomatic (Table 2, quote 1.1c). Route of administration also played a role: oral therapy was generally considered the most acceptable, although large Pentasa tablets and granules were noted as difficult to swallow and unpleasant in taste (Table 2, quote 1.1d).

Subcutaneous and rectal therapies were considered as higher risk for non-adherence due to unfamiliarity or administration difficulties. Complex regimens, high pill burdens or polypharmacy, especially without imminent clinic appointments, were further barriers. Conversely, clear medication benefits, such as symptom relief and improved QoL, with previous positive experiences of treatment, strongly encouraged adherence (Table 2, quote 1.1e).

Lifestyle and social circumstances (eg, busy schedules, travel, disorganization, or homelessness) could disrupt medication routines (Table 2, quote 1.1f). Limited treatment access, treatment storage difficulties, or high treatment costs added further obstacles.

Overall, while most HCPs believed their patients were generally adherent, they emphasized that adherence was fluid, situational, and influenced by the interplay of personal, clinical, and social factors (Table 2, quotes 1.1a–f).

##### Adherence Honesty

People tell me they take their medications, but I know that in reality a lot of people don’t take their medication. So, I don’t know if people are always being 100% honest with me about taking it. (P6, F, IBD Nurse Specialist)

HCPs reflected on the challenges of ensuring patient honesty around adherence.

Most patients were perceived as generally honest, though one consultant estimated that approximately 15% were “honest non-adherers”. Others noted a mismatch between patient behavior and reported adherence, observing that those who readily agreed to everything were not always fully truthful. Several HCPs felt that patients were not deliberately lying but wished to please their clinician, whilst avoiding their disappointment. By contrast, patients who actively questioned or took ownership of their treatment were viewed as more likely to be candid. A psychiatrist suggested it was good practice to assume all patients might, at times, be non-adherent, as this expectation could create an open environment in which honesty was more likely.

Dishonesty was considered most likely when patients felt guilty about non-adherence (Table 2, quote 1.2) or feared being judged or reprimanded by their HCP (Table 2, quote 1.2a).

Consultants in particular emphasized the importance of honesty, describing it as essential to avoid wasted time, reduce misaligned expectations, and inform safe and appropriate prescribing decisions (Table 2, quote 1.2b).

Several HCPs reported that they sought to reassure patients that they had the right to choose not to adhere but stressed the importance of being open about such decisions. Transparency was seen to facilitate frank discussions about the risks of non-adherence and to support collaborative decision-making (Table 2, quote 1.2c).

Adherence honesty was regarded as fundamental to foster long-term, high-quality relationships between patients and HCPs while also ensuring safe clinical practice (Table 2, quote 1.2d).

##### Patient Sub-Groups

You can lead a horse to water, but you can’t make them drink and we’ve done everything for them and they’ve just not turned up or they’ve not engaged. (P16, F, Consultant Gastroenterologist)

HCPs identified several patient sub-groups perceived to be at greater risk of non-adherence.

Younger patients were most frequently described as non-adherent, often seeking greater autonomy in treatment decisions (Table 2, quote 1.3a). Transitioning from pediatric to adult services was viewed as a particularly high-risk period, with developmental and social changes disrupting routines (Table 2, quote 1.3b). By contrast, older patients were generally seen as more compliant and engaged, though non-adherence could arise from polypharmacy, comorbidities, or forgetfulness, particularly in those living alone (Table 2, quotes 1.3c-d).

Low health literacy, limited education, and financial constraints were considered key barriers. Some patients reportedly missed infusions to avoid loss of earnings, while certain occupational groups, such as farmers, were seen as less likely to adhere (Table 2, quote 1.3e).

Younger men were described as the least adherent, influenced by embarrassment, denial of illness, and a desire to appear “normal”. One HCP referred to this group as the “naughty boys’ club” (Table 2, quote 1.3f). Middle-aged men, particularly if single, were perceived as more forgetful and disorganized. Women, by contrast, were more likely to express concerns about fertility, pregnancy, and appearance but were considered more open to rectal therapies and psychological support, factors which could promote adherence.

Ethnic and religious beliefs were frequently cited as influencing adherence, for example reluctance among some Muslim women to use rectal treatments. Language barriers were described as contributing to misunderstanding and miscommunication.

Across professions, HCPs voiced frustration with a small subgroup who remained disengaged despite support (Table 2, quote 1.3g), often illustrated with the repeated phrase “you can lead a horse to water…” (Table 2, quote 1.3). Some questioned whether these patients were fully informed, suggesting that more comprehensive discussions might help address gaps in understanding.

##### Language with Patients

I don’t think I’d use any of those words to patients themselves. They are my words. They’re our words, not their words. (P9, F, Nurse Consultant)

HCPs were very conscious of the language they used when discussing medication and treatment programmes with patients, aiming to avoid judgement or blame, particularly in conversations about non-adherence. Most preferred simple, clear, and transparent “patient-level” language that they felt patients would easily understand. Terms such as adherence, compliance and concordance were rarely used directly with patients, as they were not considered meaningful or relatable (Table 2, quotes 1.4, 1.4a-b). They had a range of what they felt were “patient-facing” alternatives, including medication taking.

While these terms were recognized within professional discourse, HCPs held differing views on their value. Compliance was widely regarded as outdated and paternalistic. Adherence was seen as the most acceptable of the three, though often avoided in patient interactions. Concordance was rarely used, perceived as poorly understood by both patients and some HCPs; however, for those who did use it, the term reflected partnership and shared decision-making rather than authoritarian practice (Table 2, quote 1.4c).

Several consultants and pharmacists disagreed that all three terms carried negative connotations, implying patients were “behaving badly”. As such, they deliberately avoided their use in clinical encounters (Table 2, quotes 1.4d-e).

#### Theme 2: What is Done by HCPs to Promote Adherence?

Methotrexate Monday, Folic acid Friday. (P16, F, Consultant Gastroenterologist)

This theme explored how HCPs sought to promote adherence to IBD medication in clinical practice. Four interrelated sub-themes were identified.

##### Empathic Care and Reassurance

They’re much more compliant if we work with them rather than against them. (P5, F, IBD Nurse Specialist)

A key sub-theme was the importance of empathy, reassurance, and personalized emotional support in promoting adherence. HCPs consistently emphasized recognizing each patient as an individual, with one noting, “patients don’t fit into boxes” (Table 2, quote 2.1a). Respecting patient choice and autonomy by offering treatment options that aligned with individual preferences were viewed as critical. HCPs saw their role as one of compassionately guiding patients and supporting informed decisions (Table 2, quote 2.1b).

Sharing responsibility for care and collaborating flexibly in line with patient goals were regarded as key to sustaining adherence. Developing long-term relationships was seen as particularly valuable in the context of chronic disease, enabling trust and rapport to build over time. Such trust was thought to increase the likelihood that patients would feel comfortable disclosing concerns or non-adherence, with nurses most often highlighting this dimension of care (Table 2, quote 2.1c).

Active listening was frequently described as a strategy to understand patient’s perspectives on treatment, daily life and challenges. Accessible support, particularly at diagnosis, was considered essential. Examples included open-door policies, helplines, proactive outreach, and regular monitoring (Table 2, quote 2.1d). Providing multiple avenues of contact was seen as important to ensure equitable access to care. Conversely, miscommunication or mismatched perceptions between patients and HCPs were viewed as barriers that could undermine treatment engagement and adherence.

##### Bidirectional Information

No old-style leaflets no. If I want someone to look at a leaflet about Adalimumab or Azathioprine or Vedolizumab or Ustekinumab I’ll say look at the CCUK web, just Google Ustekinumab CCUK. (P1, M, Consultant Gastroenterologist)

Information provision, both at diagnosis and when discussing medication options, was considered as a core aspect of care, enabling patient understanding and informed decision-making. HCPs typically began by outlining key facts about treatment purpose, mechanism, and duration, supported by written resources or online materials. Many directed patients to the CCUK website as a trusted, accessible source that patients could revisit in their own time (Table 2, quote 2.2).

Risks, side-effects and benefits of treatment were discussed openly and transparently, often including the consequences of non-adherence. Several HCPs found analogies, anecdotes and quantified statistics helpful in clarifying risks and providing reassurance (Table 2, quotes 2.2a-b). Framing relative risk in this way was seen to strengthen patients’ confidence in both treatment rationale and the professional capacity of their HCP, promoting informed engagement. Patients were actively encouraged to ask questions and share concerns, with HCPs describing this as an opportunity to troubleshoot collaboratively (Table 2, quote 2.2c).

However, views diverged on the extent of information appropriate at diagnosis. Some HCPs, particularly female gastroenterologists, were reluctant to inform patients that medication would necessarily be life-long, as this was not always the case (Table 2, quote 2.2d). Nurses, in particular described presenting treatment as a “trial” to reduce the perceived pressure of starting therapy, while gradually building patient trust (Table 2, quote 2.2e). Overall, bidirectional information sharing was strongly favored, both to foster confidence in treatment choices and to support more accurate assessment of adherence (Table 2, quotes 2.2f-g).

##### Practical Tips and Advice

Alarms or the [medication] apps… Physically putting it somewhere, stick it in your knicker drawer or where your keys are - so that you literally have to trip over it. (P24, F, Gastroenterology Dietician)

HCPs discussed a range of practical strategies, both implemented in clinical practice and recommended to patients, to support medication adherence. Flexible and feasible treatment regimens, such as once-daily, weekly, or monthly schedules, were considered particularly effective, as their simplicity made them easier to integrate into daily life (Table 2, quote 2.3a). Personalizing medication plans to fit individual lifestyles was emphasized, with nurses often providing supervised training and demonstrations to build confidence in treatment administration (Table 2, quote 2.3b).

Memory aids, including alarms, phone apps and visual prompts, were frequently suggested for patients who regularly forgot their medication. Linking medication-taking to daily routine was also encouraged, helping to establish it as a habit (Table 2, quote 2.3). Family members or friends were sometimes enlisted as external reminders, as well as providing practical assistance with self-injection and emotional support for continued adherence (Table 2, quote 2.3c).

Organizational tools were also widely recommended, including diaries, calendars, post-it notes, and dosette boxes, with the latter also serving a storage function (Table 2, quote 2.3d). However, dosette boxes were acknowledged as unsuitable for certain medications or patients, with some gastroenterologists noting that younger people in particular might find them unacceptable. In such cases, more modern or creative alternatives were suggested, such as wearable reminders or memorable acronyms, for example, “Methotrexate Monday, Folic acid Friday”, (P16, F, Consultant Gastroenterologist) (Table 2, Title-theme 2 quote).

##### Clinical Duties and Responsibilities

There’s a duty of care you need to let them know what you think the likely outcome and possible worst outcome that might be. (P13, F, Gastroenterology Specialist Registrar)

HCPs highlighted their shared responsibilities within the multidisciplinary team (MDT) to provide holistic and collaborative care in support of adherence. Presenting a united front, maintaining clear roles, and drawing on complementary expertise were regarded as fundamental to effective practice.

Ongoing monitoring and follow-up, including updates, risk assessments, and therapeutic drug monitoring (TDM), were considered essential for guiding treatment adjustments and supporting adherence. However, patients were sometimes perceived to undervalue monitoring, particularly when asymptomatic, underscoring the need for reinforcement of its importance (Table 2, quote 2.4a).

Balancing evidence-based prescribing protocols with personalized care was described as a constant challenge. Standardized formularies and treatment algorithms provided structure, but HCPs stressed the importance of tailoring regimens with compassion while also considering medication costs. Sharedcare arrangements with General Practitioners (GPs), home-care delivery services, and community pharmacies were viewed as potentially supportive but often unreliable, creating additional barriers to adherence (Table 2, quote 2.4b).

Many HCPs described a duty of care to optimize adherence but also to manage patient expectations transparently. Clear communication regarding disease severity and the short- and long-term benefits of treatment was seen as vital to ensuring safety and informed decision-making (Table 2, quote 2.4).

Honesty, openness to questions, and accountability when treatment failed were all framed as professional responsibilities. Several HCPs also reflected on the importance of recognizing the limits of their scope of practice and engaging in refresher training to ensure that skills were applied consistently and effectively in clinical care.

#### Theme 3: Challenges for HCPs in Adherence Support?

Medicine would be really easy if you have one patient and we would all be absolutely brilliant, not just doctors, but healthcare workers, if you had more time and less patients to look after. (P18, M, Consultant Gastroenterologist)

Theme 3 focused on the challenges HCPs encountered in supporting adherence in IBD, encompassing everyday service pressures, adherence specific barriers, and difficulties arising from their own practice.

##### Day-to-Day Challenges

We probably don’t have the time or resources to tackle it [adherence] as well as we could do really. I probably admit that I don’t routinely ask about adherence as well as I should do. (P21, M, Lead Pharmacist Gastroenterology)

HCPs described how adherence was often not routinely discussed with patients, largely due to heavy workloads, staff shortages, and limited MDT expertise (Table 2, Title-theme 3 quote). These systemic pressures, particularly acute for IBD nurses, meant adherence monitoring was frequently deprioritized (Table 2, quote 3.1). Lengthy waiting lists, including delays of up to a year for psychological referrals, compounded the problem, with many services lacking access to psychological support altogether (Table 2, quote 3.1a). Financial pressures and the rising cost of biologics also influenced prescribing decisions, occasionally affecting adherence when patients expressed brand preferences.

Service delivery models further shaped adherence support. Expanding waiting lists and time constraints led some services to adopt Patient Initiated Follow-up (PIFU) (Table 2, quote 3.1b). While this reduced demand, HCPs acknowledged that it risked leaving patients feeling unsupported, potentially undermining adherence.

Several HCPs emphasized that investing time with patients early in their “IBD journeys” could foster long-term engagement, with adherence discussions seen as particularly valuable at this stage (Table 2, quote 3.1c).

Rigid care pathways were also perceived as barriers, particularly when complex cases required more flexibility than resources allowed. Increasing daily queries meant many services could not respond within their specified timeframe (Table 2, quote 3.1d).

Patient-level challenges included unrealistic expectations and increased demand for care, exacerbated in some instances by post-COVID telephone rather than face to face appointments, which were seen to reduce satisfaction, hinder communication, and contribute to non-adherence (Table 2, quote 3.1e).

##### Adherence Specific Challenges

Sometimes you do think I wish we could go back 30 years where they would just be like, well, the doctor said I should do it. So, I’m just gonna do it because you’re like I know your life would be so much better. (P14, F, Consultant Gastroenterologist)

HCPs identified several challenges that related specifically to adherence. Some stemmed directly from patient-level barriers, such as needle phobia, which often went unaddressed due to limited service capacity. While the availability of multiple treatments was seen by some as an opportunity to enhance patient choice and support adherence, others felt it could encourage unnecessary switching without tackling underlying concerns (Table 2, quotes 3.2a-b).

Patients who blamed themselves for previous non-adherence were described as particularly difficult to support, especially in the absence of coordinated healthcare approaches (Table 2, quote 3.2c). Further challenges involved patients who mistrusted medication or preferred dietary supplements, and those who were asymptomatic but needed ongoing treatment (Table 2, quote 3.2d).

Managing patients perceived as “defiant”, “disengaged”, or “dishonest” was described as frustrating, particularly when non-adherence remained undetected and TDM was unsuitable for certain treatments (Table 2, quote 3.2). However, several HCPs acknowledged the tension between respecting patient choice and reinforcing the clinical importance of adherence, noting this balance was not always achievable.

HCPs also recognized that many influences on adherence lay outside the clinic. Limited treatment access, unreliable home-care provider services, and external influences from peers, family, online forums, or anecdotal reports were all seen as shaping patient behavior and beliefs (Table 2, quotes 3.2e-f).

##### Am I Part of the Problem?

I’m hoping I’m not normalizing it so much that they think it’s OK to not do it. So you sort of reinforce it, you know if you’re just missing it, you know now and again that’s OK. But obviously if you miss it regularly, it is more problematic. I don’t think I normalize it that much. (P14, F, Consultant Gastroenterologist)

Some HCPs, particularly females, reflected on how their practice might inadvertently contribute to non-adherence, with one nurse describing it as significant concern (Table 2, quote 3.3a). For many, the interview prompted reflection on their role, as they had tended to focus on avoiding judgmental or punitive approaches that framed patients as “bad” if they did not adhere (Table 2, quote 3.3b). Doctors particularly, described using normalization strategies to reduce blame and reassure patients that occasionally missing medication was “common” (Table 2, quote 3.3c). However, one consultant acknowledged the potential risk of such reassurance in undermining adherence (Table 2, quote 3.3).

A lack of empathy, particularly when patient concerns were dismissed, was seen as unhelpful. Communication gaps were also identified, including insufficient explanations about IBD and its treatments without checking patient understanding and encouraging questions. Yet the risk of overwhelming patients with excessive information during initial consultations was also recognized.

Several participants admitted that adherence was not consistently monitored or “owned” by clinicians. Addressing non-adherence was perceived as potentially difficult or confrontational, and some acknowledged making assumptions about non-adherence without objective evidence (Table 2, quote 3.3d). Others reported the opposite tendency: assuming adherence unless patients raised concerns themselves, bypassing routine monitoring (Table 2, quote 3.3e).

Many HCPs felt underprepared to support adherence. Most reported no formal training, aside from a few pharmacists who had encountered it during their professional education. Few were familiar with behavior change theories, and there was uncertainty over who should take responsibility, with psychologists often assumed to be best placed. Overall, there was consensus that much about non-adherence remains poorly understood (Table 2, quote 3.3f).

#### Theme 4: What Next to Support HCPs and Patients to Achieve Adherence?

A drug is only as good as if the patient takes it or turns up for the treatment. (P10, F, IBD Nurse Specialist)

The final theme explored strategies to enhance adherence support, with sub-themes addressing the needs of specific patient cohorts and service improvements, as identified by HCPs.

##### What’s Needed for Patients?

I think mostly when it’s an unintended choice with enough reassurance and support, you can get over that hump. (P14, F, Gastroenterology Consultant)

HCPs emphasized that patients require structured, ongoing support to maintain adherence, beginning at the start of their IBD journey (Table 2, quote 4.1a). This includes comprehensive education covering treatment benefits and risks, alongside managing realistic expectations. A range of reliable digital and non-digital resources, such as CCUK, should be offered to patients, families, and carers to facilitate self-learning and understanding (Table 2, quote 4.1b). Practical tools and interactive resources were seen as beneficial in promoting adherence. Examples included personalized blister packs, text message reminders, and “flare cards” to support self-management and empower patients (Table 2, quote 4.1c). Patient contact was recommended to be person-centered, regular, and accessible, with HCPs encouraged to build rapport through attention to personal details. The use of personal “hooks” (additional details about that patient) in correspondence was suggested by some nurses to help remember patients. “Patient meets” connecting individuals with peers who have experience with treatment were viewed as effective in alleviating concerns and fostering reassurance (Table 2, quote 4.1d). Peer support through local groups and patient experts was highlighted as a means to normalize living with IBD, reduce isolation, and share practical strategies. HCPs also stressed the importance of consistently monitoring and encouraging patient motivation, recognizing that low motivation increases non-adherence risk (Table 2, Title-Theme 4 quote). Overall, participants agreed that with sufficient support and guidance, patients are capable of achieving and sustaining adherence to their IBD medication (Table 2, quote 4.1).

##### What’s Needed for HCPs?

I think it’s probably we could just do better with asking about adherence on a routine basis. (P21, M, Lead Pharmacist Gastroenterology)

HCPs recognized that they could directly influence adherence support through more consistent approaches. Regular monitoring, including follow-up appointments, TDM, risk assessments, and adherence protocols, were considered essential. Staying informed on IBD adherence research could help reinforce the importance of prioritizing adherence support (Table 2, quote 4.2a), and a way to promote best practice and flexible, patient-centered care. Such methods were seen to enhance treatment efficacy, patient safety, and timely adjustments to therapy. HCPs emphasized the importance of integrating psychological, social, and medical care through effective multidisciplinary teamwork and communication, ensuring patients’ broader needs beyond IBD were met (Table 2, quote 4.2b). Improved shared care agreements with GPs were identified as a strategy to facilitate referrals, enhance collaborative care, and enable treatment accessibility. Many suggested that adherence support should be embedded across professional roles, for example pharmacists overseeing drug monitoring, nurses providing first-line support, and specialist teams managing complex cases. Expert HCPs could liaise with charities and pharmaceutical companies to deliver coordinated adherence education and peer-support programmes (Table 2, quote 4.2c).

Training for HCPs was widely recommended, with a preference for flexible, practical, scenario-based modules that incorporated real-world cases, behavior change techniques, and communication skills (Table 2, quote 4.2d). Barriers identified included time constraints, competing priorities, and the need to ensure training remained relevant and applicable (Table 2, quote 4.2e). Online delivery was favored for convenience, though hybrid models combining in-person sessions were suggested to reinforce learning. Short, interactive modules, potentially with incentives and multiple sessions to enable application in practice, were seen as optimal. HCPs anticipated that improved adherence resulting from such training could have a substantial impact on patient outcomes (Table 2, quote 4.2f).

##### What’s Needed for Healthcare Services?

I’m quite protective about wanting to see our patients in a specialist service so that we can give them the best service regardless of how extensive or complex their disease is. (P4, F, Advanced Clinical Nurse Practitioner)

HCPs emphasized the need for a culture shift in clinical practice to address systemic and policy barriers that impede adherence support. Maintaining IBD as a specialist service with clear referral pathways and designated adherence leads was considered essential (Table 2, quote 4.3).

Many advocated moving from a purely medical model to a holistic, partnership-driven approach, with personalized care planning in which HCPs and patients collaborate to support adherence (Table 2, quote 4.3a). However, delivering holistic care was recognized as challenging due to time constraints (Table 2, quote 4.3b). Peer review and service audits were suggested as mechanisms to strengthen adherence support and service delivery (Table 2, quote 4.3c).

HCPs proposed practical improvements, including modifications to medication form and size by pharmaceutical companies. This could enhance usability, alongside expanded tools to support treatment planning and organization (Table 2, quote 4.3d). Adjustments to prescription charges were viewed as another potential strategy to improve adherence.

Concerns were raised about home-care drug delivery systems, which can compromise continuity of care and limit adherence monitoring, increasing non-adherence risks (Table 2, quote 4.3e). HCPs expressed hope that a recent UK parliamentary review of home-care services could improve patient care and reduce pressure on the NHS.

##### Adherence Interventions?

And the bottom line is, people really like talking to people. (P18, M, Consultant Gastroenterologist)

HCPs suggested a range of interventions to support IBD medication adherence, with technology-based tools being prominent. Reminders and alarms were widely endorsed to assist with both medication-taking and attendance at blood tests or appointments (Table 2, quote 4.4a). Ideal digital tools were described as interactive, visually engaging, providing real-time personalized feedback, and accessible to both patients and clinicians. Integration within healthcare systems was considered important to prioritize patient experience over clinic throughput. However, HCPs cautioned that over-reliance on digital approaches could weaken rapport, limit long-term engagement, and exacerbate health inequalities.

Human-based interventions remained highly valued. These included in-person or telephone consultations for education and monitoring, as well as carefully facilitated peer support, particularly recommended by female HCPs, to avoid dominance by individual participants or the provision of unhelpful advice (Table 2, quotes 4.4b-c). Practical organizational strategies, comparable to dosette boxes, were also suggested to support routine medication-taking (Table 2, quote 4.4d). Some HCPs advocated for blended approaches combining digital and human interactions, allowing interventions to be tailored to individual patient preferences (Table 2, quote 4.4).

One consultant recommended multi-component, stepped-care approaches to structure adherence support in phases, analogous to the “Ready, Steady, Go” programme used in care transitions. Patients could progress from not ready, to preparing, to being ready for medication, with support adjusted accordingly (Table 2, quote 4.4e).

Overall, HCPs emphasized that flexibility is essential, as no single approach fits all patients. Success relies on matching the right intervention to the right individual, accommodating personal needs, preferences, and circumstances.

## Discussion

This study explored HCPs’ perceptions of the factors influencing adherence to IBD medications and strategies to support patients, highlighting the complex, multi-level nature of adherence. This is the first qualitative study of its kind. Aims were achieved through conducting interviews with a range of gastroenterology HCPs, offering insights into clinical practice and informed recommendations to enhance adherence in future care.

### Theoretical Background

Themes generated from the study were compared against three established theoretical approaches to better understand IBD medication adherence, whilst offering clarity and validation of findings:

1. Theoretical Domains Framework (TDF)^21^– to explain HCPs’ views on why patients are adherent or non-adherent (Figure 2).
2. COM-B model^22^– to analyze HCPs’ own behavior in supporting adherence, focusing on capability, opportunity, and motivation (Figure 2).
3. Theory of Planned Behavior (TPB)^23^– to explain patients’ adherence and non-adherence behaviors (Figure 3).

### HCPs’ Estimations of IBD Medication Adherence

This study found that HCPs’ perspectives on IBD adherence varied, with many believing that most patients were adherent. However, clinicians are known to overestimate adherence, often overlooking low or non-adherence,^5^ which can worsen disease control, increase flares, and escalate healthcare costs.^8^ Such assumptions, that patients will follow advice, may themselves act as barriers to effective adherence support.

Identifying non-adherent patients remains challenging in the absence of standardized measures.^25^ Validated tools, such as the Visual Analogue Scale (VAS) and the Morisky Medication Adherence Scale-8 (MMAS), offer practical ways of quantifying adherence and exploring underlying reasons for non-adherence.^26–28^ Since self-reports frequently overestimate adherence,^12^,^29^ additional measures, including TDM, provide valuable complementary insights. HCPs’ inaccurate prediction of non-adherence in IBD may stem from not routinely asking about medication use, often assuming adherence by default. Some clinicians described “leaving patients to it”, particularly within PIFU systems, where patients are expected to proactively seek care. While PIFU can empower patients,^30^ reducing both service pressure and carbon footprint of care,^31^ it can also leave patients feeling abandoned, increasing risk of disengagement. Patients may assume they no longer require treatment, with non-adherence going unrecognized. Previous research shows that patients rarely volunteer information about non-adherence, often describing themselves as “naughty” for not taking treatment.^19^ Indeed, more than one-third of non-adherent patients under-report or conceal the extent of missed medication.^12^ To mitigate these risks, patients on PIFU should be carefully assessed for their likelihood of non-adherence, and HCPs must be reminded that adherence typically declines over time.^32^ Routine, systematic screening for adherence at every clinical contact, rather than reliance on subjective judgment, is therefore critical.

### HCPs’ Perspectives as to Why Patients Adhere to Their IBD Medication

HCPs generally perceived adherent patients as those who were motivated, believed in the value of treatment, and were offered choice. This sense of autonomy was seen to increase acceptance of medication, which has been shown to improve adherence in IBD.^33^ The concept of “getting patients to buy-in” to take medication resonates with the TPB,^23^ which predicts that adherence is influenced by intention (motivation) and perceived behavioral control. Whether medication choice is offered is a pivotal moment, as it facilitates patients’ sense of control and enhances adherence likelihood (Figure 3).

Sustained adherence was thought to be most likely when treatment was initiated at diagnosis, was compatible with daily routines, with manageable side-effects or administration discomfort. Also, when medication was perceived as effective, accessible, and affordable. Patients have also highlighted the importance of flexible, practical regimens and understanding the necessity of treatment.^19^ Both HCPs and patients have identified personalized, empathic, and non-judgmental care as central to adherence. While most HCPs believed they provided such care, patients have frequently reported a lack of personalization and expressed low satisfaction.^19^ This reflects evidence that patient-centered approaches are inconsistently applied in practice.^34^ Effective adherence support requires open, tailored dialogue that addresses individual barriers,^26^ rather than a “one size fits all” approach, and must take account of the whole patient.

Notably, HCPs and patients diverge in their views on the influence of diagnostic experiences. HCPs often felt that more challenging diagnostic journeys promoted adherence, whereas many patients disagree. Instead, patients have described avoiding medication when unwell, when experiencing side-effects, or when struggling with large tablets, particularly during flares.^19^ These barriers were often unrecognized by HCPs, who assumed oral medication became “second nature”. This gap reflects a broader trend of HCPs underestimating the impact of symptoms and patient priorities.^12^ As the patient–clinician relationship is considered the most important determinant of adherence,^9^ strengthening this through direct, non-judgmental communication may help close perception gaps and reduce non-adherence.^35^

### HCPs’ Perspective on Why Patients Do Not Adhere to Their IBD Medication

HCPs identified certain sub-groups as being at greater risk of non-adherence, including younger individuals, men, those who were single or lacked social support and, in some cases, older adults. These perceptions are supported by recent reviews reporting young age, male sex, and single status as common risk factors for non-adherence.^26^ A recurring concern among HCPs was the influence of others on patients’ adherence behaviors. Younger patients particularly, were thought to be strongly shaped by family, friends, or online peers. Within the TPB^23^ and TDF^21^ frameworks, these reflect subjective norms^23^ or social influences,^21^ which can discourage adherence in chronic conditions when external advice conflicts with medical recommendations^36^ (Figures 2 and 3). In response, HCPs and prior research,^19^,^37^ have highlighted the value of peer support and patient groups (“patient meets”) in fostering adherence. Shared experiences were often perceived as more persuasive than HCP advice, helping to normalize IBD and reduce feelings of isolation.^19^ Evidence indicates that peer support can improve adherence cost-effectively across chronic conditions,^38^ with emerging but limited data in IBD, primarily among younger patients.^39^ Further research is needed to establish its effectiveness in adults with IBD.

Despite recognizing strategies to enhance adherence, many HCPs expressed skepticism that some patients could ever be supported effectively, likening it to the adage: “You can lead a horse to water, but you can’t make them drink”. This contrasts with earlier findings where 96% of interviewed clinicians believed non-adherence could be addressed.^6^ Supporting autonomy, incorporating patient preferences, and adopting collaborative approaches were nonetheless regarded as critical to improving adherence, aligning with previous research and patient perspectives.^6^,^12^,^19^,^35^

HCPs reported efforts to communicate using lay language, yet patients have often felt under-informed and described needing to seek information independently.^19^ According to World Health Organization prescribing guidelines,^40^ HCPs should ensure patients initiating treatment receive clear, accessible information and opportunities to ask questions, raise concerns, and access recommended resources.

In practice, HCPs frequently suggested interactive electronic interventions, such as reminders, calendars, and mobile apps with tailored features. Tools offering bi-directional communication between patients and clinicians and integrating with healthcare systems were considered especially valuable. Evidence supports the use of patient-centered digital technologies to improve adherence in IBD,^26^ though HCPs cautioned that interventions must consider patients’ differing needs, preferences, and digital literacy.

### Challenges to Supporting Medication Adherence

The TDF^21^ and COM-B^22^ models provide useful frameworks for understanding the barriers HCPs face in supporting adherence, whether arising from the realities of everyday practice or adherence-specific challenges.

Time pressures, workload, and competing priorities were the most frequently cited barriers, also recognized by people with IBD.^19^ Structured consultations supported by validated questionnaires could help overcome these limitations. This could enhance patients’ capability whilst offering HCPs opportunities to systematically address adherence,^29^ aligning with COM-B.^22^ For example, the DIALOG tool in mental health has shown how structured patient responses can effectively guide consultations.^41^

Financial barriers were also acknowledged. Some HCPs felt that prescription charges may deter adherence, a view echoed by patients.^19^ This aligns with the TDF^21^ and the domain of requiring resources to take medications as prescribed (Figure 2). Evidence suggests that people living in socially deprived areas are more likely to be non-adherent due to cost, facing a five-fold higher risk of disease relapse.^42^

Service-level barriers were prominent, with both HCPs and patients reporting concerns about home-care services. Delays, errors, and avoidable harm have been highlighted in a recent UK parliamentary review, which called for greater transparency, accountability, and reform.^43^ Fragmentation of care due to shared-care arrangements and limited psychological support further hindered adherence monitoring and consistency. While many HCPs acknowledged responsibility for supporting adherence, some research suggests that non-adherence is perceived as “everybody’s problem, but nobody’s responsibility”.^29^ This can be linked to the TDF^21^ domain of professional role and identity as an impact on HCPs’ behavior. In practice, adherence support is often deprioritised.^10^ Interviewed HCPs emphasized that adherence support should be a shared responsibility across the MDT, ensuring holistic and collaborative care. Pharmacy-led interventions offer promise, balancing structured workflows, patient engagement, and effective adherence strategies.^34^

Another key challenge was the difficulty addressing non-adherence directly. HCPs reported avoiding conversations that could become awkward or confrontational, and all noted a lack of training in this area. Similar findings in previous research show that clinicians’ knowledge of IBD self-management is suboptimal^44^ and that HCP training on adherence support is limited.^34^ Within the TDF^21^ and COM-B^22^ frameworks, these barriers reflect gaps in knowledge, skills, and perceived capability. Improving training for doctors, pharmacists and other MDT members in patient-centered, concordant approaches could strengthen their capability and opportunity to provide effective support.^34^ HCPs also highlighted the importance of staying updated with evidence-based research, consistent with earlier findings,^10^ aligning with knowledge and skills development in the TDF domains.^21^,^22^

Adherence support was seen as particularly crucial at diagnosis. HCPs recommended open-door policies, helplines, monitoring, and proactive outreach at this stage, recognizing that newly diagnosed patients often have limited understanding of IBD or the importance of long-term medication use. Early intervention is essential, as patients may struggle with denial or resistance to treatment in response to the psychological impact of a chronic disease.^26^,^45^,^46^ Evidence confirms that addressing barriers during this critical period improves both initial and sustained adherence.^11^

### Strengths and Limitations

This study systematically explored barriers and facilitators to IBD medication adherence from HCPs’ perspectives. Online, charity-led recruitment enabled participation across a range of professions and clinical experience. Virtual interviews enhanced accessibility and ensured consistency through a single interviewer. Application of established frameworks, including the TDF^21^ and COM-B,^22^ strengthened analysis by linking HCP beliefs and behaviors to capability, opportunity, and motivation. This approach supported interpretation and development of practical recommendations for targeted adherence interventions.

Limitations include potential self-selection bias, as those interested in adherence may have been more likely to participate, and reliance on single timepoint interviews, could be affected by recall bias. Longitudinal qualitative research may better capture how adherence support evolves in practice.

While the TDF^21^ is widely used, it lacks formal guidance^47^ and is more commonly applied prospectively than retrospectively.^48^ Developing clearer, adaptable guidelines for different settings and populations would broaden the framework’s utility.^49^

The TPB^23^ also has limitations; its linear structure does not fully capture the dynamic nature of adherence, where intention does not always translate into behavior, highlighting an intention–behavior gap.^50^

## Conclusions

HCPs recognized multiple factors influencing IBD medication adherence and are well-positioned to support patients through compassionate, tailored, and holistic healthcare. Clear, accessible information and guidance can encourage patients’ involvement in their care.

Barriers to adherence support span patient, treatments, healthcare systems, and HCP practices. Acknowledging the influence of their own behaviors and practice limitations is an important step for HCPs in optimizing adherence.

At service-level regular training, auditing, peer review, policy development and multidisciplinary collaboration, together with integration of adherence monitoring in routine care are essential to prioritize adherence and support better outcomes in IBD.

## Data Availability

All data collected for the purposes of this study are handled and stored in accordance with the United Kingdom’s (UK’s) General Data Protection Regulation (UK GDPR) and the UK’s Data Protection Act 2018. The data underlying this article are available in the article and in its online Supplementary data. Identifying information about the participants were removed from the data. Each participant was assigned a unique participant code, which was used on all of their data. A separate document that links the study codes to the identifying information has been digitally stored and protected. Only the research team had access to this document.

## Abbreviations

CCUK, Crohn’s and Colitis UK; CD, Crohn’s disease, G.P, General practitioner/Family doctor; F, Female; IBD, inflammatory bowel disease, HCP, Healthcare professional; IBDU, inflammatory bowel disease unclassified; M, Male; MDT, Multidisciplinary teams; NHS, National Health Service; P, Participant; PIFU, Patient initiated follow-up; RTA, Reflexive thematic analysis; TDF, Theoretical Domains Framework; TDM, Therapeutic drug monitoring; TPB, Theory of Planned Behavior; UC, Ulcerative colitis; UK, United Kingdom.

## References

[1] King D, Reulen RC, Thomas T, et al. Changing patterns in the epidemiology and outcomes of inflammatory bowel disease in the United Kingdom: 2000-2018. *Aliment Pharmacol Ther*. 2020;51(10):922–27. doi:10.1111/apt.15701 32237083

[2] Burisch J, Kiudelis G, Kupcinskas L, et al. Natural disease course of Crohn’s disease during the first 5 years after diagnosis in a European population-based inception cohort: an Epi-IBD study. *Gut*. 2019;68(3):423–433. doi:10.1136/gutjnl-2017-315568 29363534

[3] Taylor CC, Millien VO, Hou JK, Massarweh NN. Association between inflammatory bowel disease and colorectal cancer stage of disease and survival. *J Surg Res*. 2020;247:77–85. doi:10.1016/j.jss.2019.10.040 31767275

[4] King K, McGuinness S, Watson N, Norton C, Chalder T, Czuber-Dochan W. What do we know about medication adherence interventions in inflammatory bowel disease, multiple sclerosis and rheumatoid arthritis? A scoping review of randomised controlled trials. *PPA*. 2023;17:3265–3303. doi:10.2147/PPA.S424024 38111690 PMC10725835

[5] Trindade AJ, Ehrlich A, Kornbluth A, Ullman TA. Are your patients taking their medicine? Validation of a new adherence scale in patients with inflammatory bowel disease and comparison with physician perception of adherence. *Inflamm Bowel Dis*. 2011;17(2):599–604. doi:10.1002/ibd.21310 20848512

[6] Soobraty A, Boughdady S, Selinger CP. Current practice and clinicians’ perception of medication non-adherence in patients with inflammatory bowel disease: a survey of 98 clinicians. *WJGPT*. 2017;8(1):67. doi:10.4292/wjgpt.v8.i1.67 28217376 PMC5292608

[7] Kane S, Huo D, Aikens J, Hanauer S. Medication nonadherence and the outcomes of patients with quiescent ulcerative colitis. *Am J Med*. 2003;114(1):39–43. 12543288 10.1016/s0002-9343(02)01383-9

[8] Kane SV. Systematic review: adherence issues in the treatment of ulcerative colitis. *Aliment Pharmacol Ther*. 2006;23(5):577–585. 16480396 10.1111/j.1365-2036.2006.02809.x

[9] Trindade AJ, Morisky DE, Ehrlich AC, et al. Current practice and perception of screening for medication adherence in inflammatory bowel disease. *J Clin Gastroenterol*. 2011;45(10):878–882. doi:10.1097/MCG.0b013e3182192207 21555953 PMC3156931

[10] Kanazaki R, Smith B, Girgis A, Connor SJ. Clinician adherence to inflammatory bowel disease guidelines: results of a qualitative study of barriers and enablers. *Crohns Colitis 360*. 2023;5(3):otac018. 37180282 10.1093/crocol/otac018 PMC10174629

[11] King K, Czuber-Dochan W, Chalder T, Norton C. Medication non-adherence in inflammatory bowel disease: a systematic review identifying risk factors and opportunities for intervention. *Pharmacy*. 2025;13(1):21. doi:10.3390/pharmacy13010021 39998019 PMC11859822

[12] Schreiber S, Panés J, Louis E, Holley D, Buch M, Paridaens K. Perception gaps between patients with ulcerative colitis and healthcare professionals: an online survey. *BMC Gastroenterol*. 2012;12(1). doi:10.1186/1471-230x-12-108 PMC3523079 22894661

[13] Improving inclusion of under-served groups in clinical research: guidance from INCLUDE project. NIHR. Available from: https://www.nihr.ac.uk/improving-inclusion-under-served-groups-clinical-research-guidance-include-project. Accessed July 23, 2025.

[14] Hennink MM, Kaiser BN, Marconi VC. Code saturation versus meaning saturation: how many interviews are enough? *Qual Health Res*. 2017;27(4):591–608. doi:10.1177/1049732316665344 27670770 PMC9359070

[15] Johnson JL, Adkins D, Chauvin S. A review of the quality indicators of rigor in qualitative research. *Am J Pharm Educ*. 2020;84(1):7120. doi:10.5688/ajpe7120 32292186 PMC7055404

[16] Braun V, Clarke V. Reflecting on reflexive thematic analysis. *Qual Res Sport Exerc Health*. 2019;11(4):589–597. doi:10.1080/2159676x.2019.1628806

[17] Boyatzis RE. *Transforming Qualitative Information: Thematic Analysis and Code Development*. Nachdr. Sage; 2010.

[18] Thapwong P, Norton C, Rowland E, Czuber-Dochan W. Our Life Is a Rollercoaster! A Qualitative Phenomenological Study Exploring the Impact of IBD on Family Members. *Inflamm Bowel Dis*. 2024;30(12):2395–2404. doi:10.1093/ibd/izae028 38417051 PMC11630016

[19] King K, Norton C, Jammeh A, Chalder T, Czuber-Dochan W. “I Probably Am Being a Naughty Boy, But…” reasons for non-adherence to prescribed medication, as perceived by people living with inflammatory bowel disease: a qualitative study. *PPA*. 2025;19:2391–2415. doi:10.2147/PPA.S531675 40860606 PMC12371128

[20] Braun V, Clarke V. Using thematic analysis in psychology. *Qual Res Psychol*. 2006;3(2):77–101. doi:10.1191/1478088706qp063oa

[21] Michie S. Making psychological theory useful for implementing evidence based practice: a consensus approach. *Qual Saf Health Care*. 2005;14(1):26–33. doi:10.1136/qshc.2004.011155 15692000 PMC1743963

[22] Michie S, Van Stralen MM, West R. The behaviour change wheel: a new method for characterising and designing behaviour change interventions. *Implementation Sci*. 2011;6(1):42. doi:10.1186/1748-5908-6-42 PMC3096582 21513547

[23] Ajzen I. The theory of planned behavior. *Organ Behav Hum Decis Process*. 1991;50(2):179–211. doi:10.1016/0749-5978(91)90020-t

[24] World Medical Association Declaration of Helsinki: ethical principles for medical research involving human subjects. *JAMA*. 2013;310(20):2191. doi:10.1001/jama.2013.281053 24141714

[25] Kane SV, Robinson A. Review article: understanding adherence to medication in ulcerative colitis – innovative thinking and evolving concepts. *Aliment Pharmacol Ther*. 2010;32(9):1051–1058. doi:10.1111/j.1365-2036.2010.04445.x 20815833

[26] Dal Buono A, Armuzzi A, Caprioli F, et al. Therapeutic adherence in inflammatory bowel disease: user guide from a multidisciplinary modified Delphi consensus. *Digestive Liver Dis*. 2025;57(7):1403–1410. doi:10.1016/j.dld.2025.04.032 40414777

[27] Severs M, Zuithoff PNPA, Mangen MJJ, et al. Assessing Self-reported Medication Adherence in Inflammatory Bowel Disease: a Comparison of Tools. *Inflamm Bowel Dis*. 2016;22(9):2158–2164. doi:10.1097/MIB.0000000000000853 27482979

[28] Williams AB, Amico KR, Bova C, Womack JA. A proposal for quality standards for measuring medication adherence in research. *AIDS Behav*. 2013;17(1):284–297. doi:10.1007/s10461-012-0172-7 22407465 PMC3434290

[29] Chan AHY, Wright DFB. Medication adherence—Everybody’s problem but nobody’s responsibility? *Br J Clin Pharmacol*. 2025;91(3):681–683. doi:10.1111/bcp.16384 39734279

[30] Morris K, Gudgeon E, Stammers M, et al. P333 Impact of IBD remote monitoring introducing a patient-initiated follow-up (PIFU) model. *Gut*. 2025;74:A294. doi:10.1136/gutjnl-2025-BSG.466

[31] Sebastian S, Whitehead E, Schranz J, Alaghband N, Parkes G. O18 Reduction in carbon footprint though a patient-initiated follow-up (PIFU) inflammatory bowel disease pathway. *Gut*. 2024;73(Suppl 1):A10. doi:10.1136/gutjnl-2024-BSG.18

[32] Carmody JK, Plevinsky J, Peugh JL, et al. Longitudinal non-adherence predicts treatment escalation in paediatric ulcerative colitis. *Aliment Pharmacol Ther*. 2019;50(8):911–918. doi:10.1111/apt.15445 31373712 PMC8215554

[33] Tiao DK, Chan W, Jeganathan J, et al. Inflammatory bowel disease pharmacist adherence counseling improves medication adherence in Crohn’s disease and ulcerative colitis. *Inflamm Bowel Dis*. 2017;23(8):1257–1261. doi:10.1097/MIB.0000000000001194 28719539

[34] Andersson M, Garfield S, Eliasson L, Jackson C, Raynor T. Delivery of patient adherence support: a systematic review of the role of pharmacists and doctors. *PI*. 2014;31. doi:10.2147/pi.s4664724693532

[35] Zolnierek, Haskard KB, DiMatteo MR. Physician communication and patient adherence to treatment: a meta-analysis. *Med Care*. 2009;47(8):826–834. doi:10.1097/mlr.0b013e31819a5acc 19584762 PMC2728700

[36] Rich A, Brandes K, Mullan B, Hagger MS. Theory of planned behavior and adherence in chronic illness: a meta-analysis. *J Behav Med*. 2015;38(4):673–688. doi:10.1007/s10865-015-9644-3 25994095

[37] Franco FCZ, de Oliveira MCC, Gaburri PD, Franco DCZ, Chebli JMF. High prevalence of non-adherence to ulcerative colitis therapy in remission: knowing the problem to prevent loss. *Arquivos de Gastroenterologia*. 2022;59(1):40–46. 35442335 10.1590/S0004-2803.202200001-08

[38] Wiemann CM, Graham SC, Garland BH, et al. Development of a Group-based, peer-mentor intervention to promote disease self-management skills among youth with chronic medical conditions. *J Pediatric Nurs*. 2019;48:1–9. doi:10.1016/j.pedn.2019.05.013 31195183

[39] Dave S, Bugwadia A, Kohut SA, Reed S, Shapiro M, Michel HK. Peer support interventions for young adults with inflammatory bowel diseases. *Health Care Transit*. 2023;1:100018. doi:10.1016/j.hctj.2023.100018 39713010 PMC11657158

[40] World Health Organization. Guide to good prescribing: a practical manual. World Health Organization; 1994. Available from: https://iris.who.int/bitstream/handle/10665/59001/WHO_DAP_94.11.pdf. Accessed July 23, 2025.

[41] DIALOG+. East London NHS Foundation Trust. Available from: https://www.elft.nhs.uk/dialog. Accessed July 23, 2025.

[42] Young people with ulcerative colitis at risk due to low adherence to prescribed medication. NIHR Imperial Biomedical Research Centre. Available from: https://imperialbrc.nihr.ac.uk/2023/09/18/young-people-with-ulcerative-colitis-at-risk-due-to-low-adherence-to-prescribed-medication/. Accessed July 23, 2025.

[43] Lords Committee publishes new report on services that could transform patient care. Committees - UK Parliament. Available from: https://committees.parliament.uk/committee/430/public-services-committee/news/198458/lords-committee-publishes-new-report-on-services-that-could-transform-patient-care/. Accessed July 23, 2025.

[44] Prasad SS, Keely S, Talley NJ, et al. Primary care pharmacists’ knowledge and perception of Inflammatory Bowel Disease: a cross-sectional study in Australia. *HEPJ*. 2022;5(1):15437. doi:10.33966/hepj.5.1.15437

[45] van der Have M, Oldenburg B, Kaptein AA, et al. Non-adherence to Anti-TNF therapy is associated with illness perceptions and clinical outcomes in outpatients with inflammatory bowel disease: results from a prospective multicentre study. *J Crohns Colitis*. 2016;10(5):549–555. doi:10.1093/ecco-jcc/jjw002 26738757 PMC4957450

[46] Chapman S, Frostholm L, Chalder T, et al. Preventing medication nonadherence: a framework for interventions to support early engagement with treatment. *Health Psychol Rev*. 2024;18(4):884–898. doi:10.1080/17437199.2024.2385525 39101263

[47] Atkins L, Francis J, Islam R, et al. A guide to using the Theoretical Domains Framework of behaviour change to investigate implementation problems. *Implementation Sci*. 2017;12(1). doi:10.1186/s13012-017-0605-9 PMC5480145 28637486

[48] Duncan EM, Francis JJ, Johnston M, et al. Learning curves, taking instructions, and patient safety: using a theoretical domains framework in an interview study to investigate prescribing errors among trainee doctors. *Implementation Sci*. 2012;7(1). doi:10.1186/1748-5908-7-86 PMC3546877 22967756

[49] Cane J, O’Connor D, Michie S. Validation of the theoretical domains framework for use in behaviour change and implementation research. *Implementation Sci*. 2012;7(1). doi:10.1186/1748-5908-7-37 PMC3483008 22530986

[50] Sniehotta FF, Presseau J, Araújo-Soares V. Time to retire the theory of planned behaviour. *Health Psychol Rev*. 2014;8(1):1–7. doi:10.1080/17437199.2013.869710 25053004

